# Data on external walls from a multi-objective simulation for cold climates

**DOI:** 10.1016/j.dib.2016.09.048

**Published:** 2016-10-05

**Authors:** Cristina Baglivo, Paolo Maria Congedo

**Affiliations:** Department of Engineering for Innovation, University of Salento, 73100 Lecce, Italy

**Keywords:** Precast, Wall, Envelope, ZEB, Efficiency, Multi-criteria

## Abstract

Data are related to the multi-objective optimization process applied to the building materials to obtain high energy-efficient precast walls for cold climate.

The methodology has been explained on the paper entitled “High performance precast external walls for cold climate by a multi criteria methodology” (Baglivo and Congedo, 2016) [1].

The modeFRONTIER rel.4.3 optimization tool has been used to evaluate the dynamic behaviour of the building components in accordance with the UNI EN ISO 13786:2008 and to obtain a multitude of high efficiency configurations.

The results are divided into three categories thick, thin and ultra-thin precast walls, in accordance with their thicknesses. The input data are the building materials with their thermal properties, sustainability characteristics and the supply and installation costs. The output values of the simulations are adapted to the cold climate and based on thermal properties, costs and sustainability score. Several combinations of external precast walls have been shown as optimal for cold climate.

**Specifications Table**

**Table:** 

Subject area	*Civil engineering,*
More specific subject area	*Design of external walls evaluating the thermal, economic and eco-friendly behaviour.*
Type of data	*Excel file*
How data was acquired	*Pricelist, technical data sheet, analysed and processed output data*
Data format	*Analyzed*
Experimental factors	*The building material properties have been provided by commercial data sheets. Each material, listed as input data of the analysis, has been used in the multi objective analysis.*
Experimental features	*The results from multi objective analysis have been shown. The analysis considered the thermal performance in accordance with the UNI EN ISO 13786:2008, the environmental impact and the costs.*
Data source location	*Bolzano, Italy*
Data accessibility	*Data is within this article.*

**Value of the data**

• The data show several high efficiency configurations for cold climate.
• It is useful for a first comparison with other studies conducted for other climates.
• It allows to develop new theories and new parameters to design optimal walls for all climates.

## 1 Data

The supplementary material, “Supplementary Table 1.zip”, presents a database of building materials listed with their thermal characteristics, eco sustainability score and commercial costs. Each material has been used within the multi-objective optimization process.

The constraints and objective of the simulation are presented and conducted for the three ranges of total thicknesses: thick walls (20–28 cm), thin walls (18–20 cm) and ultra-thin walls (<18 cm).

The model configuration established is a wall with five layers, in accordance with the common practice.

## 2 Experimental design, materials and methods

The calculation procedure based on the multi-objective analysis has been performed by the followed steps:

1) Creation of a database containing building materials.
2) Definition of the objectives and constraints of the simulation.
3) Calculation for the three thickness categories.

The details of the methodology are presented in [1].
